# On Diseases of the Bladder and Prostate Gland. With Plates

**Published:** 1840-04-01

**Authors:** 


### On Diseases of the Bladder and Prostate Gland. With Plates. By *William Coulson*. Second Edition, greatly enlarged, 8vo. pp. 258, four plates. Longman and Co. 1840.

We had occasion to notice, and in very favourable terms, the first edition of Mr. Coulson's work. The speedy appearance of the second, is as gratifying to us, as it must be to the author, a gentleman possessed of great zeal for his profession and well acquainted with the class of disorders to which he has devoted his attention.

The present edition is much augmented both in size and value. Mr. Coulson remarks in his preface to it:—"My chief object in the former edition, was, to establish clearly the distinction between the inflammatory diseases which attack the several coats of the bladder. It appeared to me important that these affections should not be confounded under the general character of inflammation of the bladder, as not only are they distinguished from one another by their symptoms and progress, but as each of them requires an essentially different plan of treatment.

Since the publication of my former edition, I have availed myself of the increased opportunities which have occurred to me, to follow up my investigations of the subject, and they have not in any way altered the practical conclusions which I formerly attempted to draw. I have endeavoured to render my present edition a more complete work as to the diseases of the bladder; and I have added three chapters on the affections of the prostate gland, which are so closely connected and so often confounded with those of the bladder."

Having given an ample account of the contents of the first edition, we shall only allude to the *new* matter in the second. That will be chiefly found in the first chapter (on urine); in the ninth (on spasm of the bladder); and in the thirteenth, fourteenth, and fifteenth, (on the affections of the prostate gland.) The chapter on urine we pass over, and proceed to that on:—

### Spasm of the Bladder.

Mr. Coulson contends that the bladder, being muscular, must, *ex neces-*

*sitate*, be liable to spasm. He describes, as follows, the symptoms that characterise it :—One of the foremost, he says, of these is the circumstance that the disease comes on in fits, and the attack is for the most part sudden: the character of the accompanying pain is likewise diagnostic—it is constrictive, corresponding with what is felt in other muscular organs subject to spasm. The pain is usually very violent, almost insupportable; and, during the continuance of the spasm, the peculiar function of the bladder is variously disordered, according to the particular set of fibres involved in the morbid affection. For example, if the fasciculi situated at the fundus and upper part of the bladder be the chief seat of the spasm, then it often happens that the contents of the bladder are suddenly and forcibly expelled on the first accession of the spasm: whereas, if the disease seizes on the neck of the bladder, the very reverse ensues—the urine is retained so long as the spasm persists. Mr. Coulson observes that spasm of the bladder is most likely to be confounded with inflammation of its mucous membrane, but the circumstances we are about to mention, are, he thinks, sufficient to distinguish them.

" In inflammation, the pain is constant, coming on with more of uneasiness than of positive pain, and exasperating by degrees; while in spasm, the seizure is as severe as it is sudden. In the former, the pain has the characters usual in inflammations—it is lancinating and throbbing; whereas in the latter, it is, as I have already observed, constrictive, resembling, in fact, labour-pains—for these also result from the spasmodic contraction of the muscular fibres of the womb in promoting child-birth. In both there usually is retention of urine, but the cause in each case somewhat differs; in spasm, seated about the neck of the bladder, the spastic contraction of the sphincter vesicæ prevents the urine from being evacuated; in inflammation of the same part, on the other hand, it is the tumefaction of the mucous coat and subjacent cellular membrane that is the cause of the obstruction. A distinction between spasm and inflammation affecting the bladder, may be partly deduced from the age of the patient, corroborative of other more positive indications, seeing it is the young and robust that are most liable to be attacked by inflammation, and the aged, the nervous, and debilitated that are most subject to spasm. The colour of the urine in the two cases is likewise a diagnostic; since in the former disease it is red and high-coloured, while in the latter it is watery and pale, especially if there be no organic affections, at the same time, in either the bladder, uterus, or kidney." 153.

In some unequivocal cases of inflammation of the bladder, which we have had an opportunity of witnessing, the pain was not of the lancinating or throbbing character, but was usually dull and heavy, as, we believe, the pain in inflammation of mucous membranes is disposed to be. In those cases too, spasm, at least of the neck of the bladder, was a never-failing concomitant; and it appears improbable that retention of urine should depend on tumefaction of the mucous membrane at the neck of the bladder, without spasm—so apt to occur is the latter. The difficulty is to separate the symptoms of inflammation from those of spasm, because the former is prone to give occasion to the latter. *That* is much more likely to exist without the former, than the former without *it*.

A " fit of the stone," is only a violent paroxysm of spasm, attended with great pain from the irritation of the calculus. The spasm sometimes extends to the rectum, and it expels its contents suddenly. At other times, says

Mr. Coulson, the spasm shuts up the orifices of the ureters, and thus prevents the urine secreted by the kidneys from finding its way into its natural receptacle, the bladder; in consequence of which, the ureters and pelves of the kidneys become greatly distended, accompanied by severe pain in the loins and down the thighs: but the most frequent line of the spasmodic extension is along the course of the urethra, indicated by the violent pain felt in the whole length of the passage; and there is a constant desire to void urine without the ability. We need not add that the spasm itself does not, probably, extend beyond the muscles of the bulb, the uneasiness anterior to that being sympathetic in its nature.

Violent attacks of spasm are apt to end in inflammation, or in exhaustion of the patient. The retention of the urine is, of course, a serious evil.

"It is at times sympathetic of hysteria; and when it has been so, I have known it to be mistaken for inflammation, and the treatment adopted has consequently been most pernicious: indeed, I knew one patient who, from the long-continued erroneous treatment pursued, was reduced to the utmost extremity; for, as the plan of cure was the one most certain to increase the nervous irritation, the attacks of spasm were the more frequent the longer it was continued." 155.

If, of frequent recurrence, it may terminate in paralysis.

*Treatment.*—This will necessarily vary with the cause that has given birth to it. If conjoined with inflammation, the remedies adapted for that state must be employed.

"If there be reason to believe the affection to originate in a gouty diathesis, or to owe its presence to a suppression of a paroxysm of this disease, while we endeavour to allay the symptoms affecting the bladder, we are to strive at the same time to induce the gout to make its appearance; for which purpose we should apply sinapisms to the feet, and blisters to the calves of the legs. The operation, indeed, of the latter affords, as Soemmering has already observed, a diagnostic peculiarity, from their often proving most beneficial in the removal of a morbid state of bladder, which otherwise they are so apt to induce. Among the palliatives of the symptoms affecting the bladder, I have found none more efficacious than a combination of colchicum, opium, and camphor, or hyoscyamus. As soon as we have got rid of the arthritic strangury, we are to direct our attention to the systematic treatment of the disease, to avert its recurrence." 156.

The rule, then, is to determine the cause of spasm, and, so far as possible, to remove it. For the spasm itself, opiates, by the mouth, by enema, or by suppository, are the best medicines. In all cases, the introduction of instruments ought to be sedulously shunned, unless, says Mr. Coulson, where it has become necessary to ascertain whether there be stone in the bladder or not; and unless, we would add, there is retention of urine, unconquerable by any other remedy.

Mr. Coulson observes that, for spasm of the bladder proceeding from sympathy with some contiguous organ that derives its nerves from the same source, the tinctura ferri sesquichloridi often proves of great service. The sphincter vesicæ is commonly the seat of the spasm, and with it we consequently have retention of urine. The operation, therefore, of the remedy appears to be purely antispasmodic, and its influence frequently is almost immediate. The usual mode of administering it is to give from fifteen to thirty minims every quarter of an hour until the spasm yields.

Mr. Coulson speaks favourably of a poultice containing powdered camphor, applied to the perineum—or a liniment of camphor and opium. We think such applications generally disappoint; those which contain belladonna are most effectual, yet *they* commonly fail. We have found more advantage from cupping on the perineum. We quite agree with Mr. Coulson in his reprobation of tobacco—a dangerous medicine; and we agree with him, too, in the necessity of being on the watch for inflammation.

An interesting chapter.

### AFFECTIONS OF THE PROSTATE GLAND.

1. *Acute Inflammation.*—Mr. Coulson enumerates the symptoms thus:—heat and pain in the perinæum, near the anus; frequent micturition; tenesmus vesicæ; intense scalding in making water, which is increased as the acceleratores urinæ contract to expel the last drops of urine. Evacuations from the bowels also cause great uneasiness, and there often remains a sensation as if the rectum was not completely emptied, giving rise to distressing tenesmus. If the finger be introduced into the rectum the gut feels hot, and sometimes the prostate is felt as a smooth, round, and hard body, projecting downward on the bowel, which the pressure made by the finger renders exceedingly painful. A catheter or sound, when it reaches the prostatic part of the urethra, occasions acute pain, and severe spasmodic contractions. The symptoms are aggravated in the sitting or the standing posture.

The inflammation is prone to extend to the mucous membrane of the bladder, when the urine becomes mucous, perhaps bloody. Retention of urine "frequently succeeds;" indeed we have seen no cases of inflammation of the prostate, in which there was not retention of urine, more or less.

The causes of acute prostatitis are the pressure or irritation of vesical calculi—"the morbid secretions proceeding from vesical catarrh, or from a diseased kidney, which directly or indirectly act upon the prostate, and excite inflammation either in its follicles, or in the cellular tissue that envelops them"—strictures in the urethra—the improper use of instruments, particularly of small metallic ones, (we are convinced that a large proportion of urethral, if not vesical alterations, are created by injudicious or unskilful surgery)—gonorrhœa—the use of the gum resins and injections for gonorrhœa, pushed, we may add, during the inflammatory stage—abuse of venery, or of diet—exposure to wet and cold—French writers say, strong coffee—cantharides—excessive riding.

If resolution occurs, the symptoms, of course, subside; at the same time, the liquid secreted by the follicles of the gland augments in quantity, and, mixing with the urine, in the form of a whitish or greyish viscid matter, settles at the bottom of the vessel without adhering to its sides, having a good deal the appearance of pus imperfectly elaborated; and this matter disappears in its turn.

The treatment will necessarily be antiphlogistic, and active in proportion to the acuteness of the inflammation, and the vigour of the patient. Mr. Coulson remarks that "in many cases the secretion of urine is considerably augmented. In young persons, however, it will be unnecessary to introduce a catheter, except when suppuration is about to take place, and there is retention of urine;" but, if we may trust our own observation, retention of urine, or, at all events, incomplete evacuation of the urine, is so common in cases of inflammation of the prostate, that the catheter is usually necessary. When rigors, and other indications of suppuration, present themselves, a careful examination should be made by the rectum, in connexion with inspection of the external swelling. The matter may be discharged into the urethra, or the bladder, or the rectum, or the pelvic cellular tissue, or the perineum.

"If the abscess opens into the urethra, a very copious purulent discharge instantly manifests itself from that passage: the urinary discharge is accompanied, but more frequently preceded or followed, by the evacuation of a large quantity of phlegmonous matter, the specific gravity of which is much greater than that of the urine and the vesico-prostatic mucous products.

It is desirable that the matter of the abscess should find its way neither into the rectum, the urethra, nor behind the bladder, which may be fatal, but should pass to the surface, in order to discharge its contents more freely, and heal more readily. We must not, therefore, hesitate, where fluctuation can be discovered, to make a puncture with a lancet, without waiting its presenting at the surface. The slight bleeding which ensues under such circumstances does good, and an abscess, either artificially or naturally opened in this way, heals with little difficulty; which may be further promoted by the internal use of sulphate of quinine, steel, or other tonics.

Should the disease have anticipated the operator, and the abscess have opened in the perinæum or the rectum, nothing can be done beyond maintaining the general health. Should the abscess have burst into the urethra, or at the neck of the bladder, it would be better to allow a flexible catheter to be retained in its canal, until there is reason to believe the abscess is healed, than to practise frequent introduction. Most commonly the matter gradually diminishes in quantity, and ceases entirely after a time. In other instances the discharge disappears for a time, and then comes on, and this may occur several times before the patient completely recovers." 234.

It is more consistent with the anatomy of the parts, as well as, we believe, with fact, to anticipate the discharge of prostatic abscess into the prostatic part of the urethra, than in any other direction. Deep perineal abscess is more frequently suppuration in the cellular tissue, *excited by* inflammation in the urethra or the prostate, than actual abscess *in* the latter, opening *into* the former. The very strong capsule of the prostate explains the limitation of matter within it, and the loose cellular atmosphere around the gland, the propagation of inflammation without it.

Mr. Coulson concludes by observing that ulceration of the surface of that portion of the prostate which projects into the bladder sometimes occurs. In this state, or even in that of mere enlargement, the gland is liable to bleed. This usually will cease by antiphlogistic treatment, and keeping the patient entirely in the recumbent position.

2. *Chronic Inflammation of the Gland.*—A consequence of acute inflammation, very frequent "in those who are subject to rheumatism and cutaneous eruptions."

"Sometimes, also," adds Mr. Coulson, "in feeble subjects, the antiphlogistic treatment stops the progress of the inflammation at a period of the disease when pus has already formed in the parenchyma of the prostate, but which has not effected its discharge outwards. Disseminated in the cellular tissue, or contained in several small abscesses, this purulent matter undergoes the same change as sometimes happens in the pulmonary tissue, and which is not wholly re-absorbed: it is deprived of its more liquid parts, and reduced to its elements of greatest consistence. Thus we perceive formed tubercular depositions that soon become the subjects of frequent inflammation, while each fresh attack only increases the deposition; and in doing so, necessarily augments the size of the gland." 237.

Mr. Coulson states, as another mode in which the gland is enlarged:—deposition of serosity into the cellular tissue ensues on every fresh exacerbation of the inflammation; the more liquid portions of it being removed, and the albuminous that remains becomes organised. Certainly enlargement of the prostate frequently takes place without any previous acute inflammation, indeed without *symptoms* of inflammation at all. An elderly person finds difficulty of micturition gradually come on, and, if it has lasted for any time, the surgeon detects some enlargement of the prostate. Such a case must rather be looked on as an instance of the changes incidental to old age, than necessarily inflammatory—changes produced apparently by congestion and defective circulation rather than by inflammation and by active circulation.

Mr. Coulson and other writers allude to scaly eruptions on the skin as concomitants of enlarged prostate, and would seem to imply some degree of mutual connexion. But scaly eruptions, are common attendants on old age, and are seen almost as often in females as in males. It appears to us that there is no further association between *them* and enlarged prostate, than between *it* and the other changes of advanced life.

Mr. Coulson's account of the symptoms of the disorder is succinct and sufficiently explicit. He adverts to the alterations in the canal of the urethra, produced by the changes in the prostate. The growth of its sides renders its cavity deeper, and the posterior extremity forms a projection into the bladder. This projection is owing to the enlargement of a separate lobule of the prostate gland: or a winding passage may be formed through the prostate, by alterations in the shape of its cavity; the channel being thrown to one side, frequently to the right one—a circumstance to be recollected on passing the catheter, when obstruction is felt at the neck of the bladder. This state may be discovered by an examination per rectum. It obviously is one of the cases in which an elastic catheter will pass easily without a stilet, though difficultly, or not at all, with one.

We transcribe the directions given by Mr. Coulson, in reference to the use of the catheter.

"The catheter should either be passed every six or eight hours as the irritation returns, or it should be left in the bladder. In the latter case, after the first two or three days, it is a general rule to withdraw it, and repass it occasionally. If, however, it is not easily introduced, it will be better to secure it in the bladder, allowing the water to flow through it at intervals.

The usual mode of securing the catheter, is to apply a common T bandage, of which the longitudinal band is divided up the middle, into two portions, one of which lies in each angle between the scrotum and thigh, and to which the catheter may be secured by ligatures.

Should the patient not empty his bladder when the catheter is withdrawn, it must be re-introduced, and after a few days again taken out.

In some cases of diseased prostate, the urethra being very irritable, is liable to spasm at the membranous part; and as the gum catheter on an iron stilet is then certain to bring on spasm, a flexible gum catheter should be passed skilfully, and with the slightest possible force. When the bladder is emptied, the catheter should be retained by the means already pointed out, the water being drawn off at intervals till the first symptoms go off, and till the bladder can retain the water for the usual length of time.

The following method has been found successful, in cases rendered difficult by spasm: namely, to pass a gum catheter as far as possible without a stilet, and this will probably be to the enlargement of the prostate; then to introduce a stilet into the catheter, without withdrawing the latter from the urethra; and, finally, to employ the stilet to direct the point over the tumour of the prostate.

In thus using the gum catheter in a flexible state, if it do not pass readily, owing to its having been softened and lost its curve by passing through the straighter part of the urethra, it should be withdrawn, and another employed." 244.

Mr. Hey pointed out another mode of using the catheter, which has now and then succeeded. The catheter is to be passed with the stilet to the enlarged prostate—then the stilet is to be partially withdrawn, a manœuvre which tilts up the end of the catheter, and may enable it to ride over the protuberant gland.

If puncturing the bladder becomes necessary, Mr. Coulson prefers doing so from the rectum. He is of opinion that when painful sensations occur in the rectum, of which the symptoms are, distress in going to stool, painful tenesmus increased by efforts, or, when exertions are no longer made, and there is an aching uneasy feeling in the parts, the best applications are suppositories of extract of hemlock, with or without the extract of opium, and glysters of warm water, as a tepid bath, and as a means of procuring regular evacuations with little disturbance.

We think this edition much improved, and a valuable accession to the existing works on urinary diseases. It goes to confirm the favourable manner in which we have, on more than one occasion, had reason to speak of its judicious and indefatigable author. We recommend it to our surgical readers.

